# Countering the threatening surge in SARS-CoV-2 variants in China and its possible global implications: stocktaking in the ongoing FISU and the Asian Games vigilance

**DOI:** 10.1097/MS9.0000000000001811

**Published:** 2024-02-07

**Authors:** Snehasish Mishra, Ranjan K. Mohapatra, Gaganendu Dash, Aroop Mohanty, Ranjit Sah

**Affiliations:** a School of Biotechnology, KIIT Deemed-to-be University, Bhubaneswar; b Department of Chemistry, Government College of Engineering, Keonjhar, Odisha; c KIIT School of Sports, KIIT Deemed-to-be University, Bhubaneswar; d Department of Clinical Microbiology, AIIMS Gorakhpur, Uttar Pradesh; e Department of Microbiology, Tribhuvan University Teaching Hospital, Institute of Medicine, Kathmandu, Nepal; f Department of Microbiology, Dr. D. Y. Patil Medical College, Hospital and Research Centre, Dr. D. Y. Patil Vidyapeeth, Pune Maharashtra, India

Highlights

- The mega continental event, the 19th Asian Games Hangzhou 2022, was shifted from 2022 to 2023 due to rapidly spreading COVID-19, similar to the fate of the FISU University Games.
- The Asian Games 2023 will ensue about a couple of months later, from 23 September to 8 October 2023, at the Zhejiang University of Technology, Hangzhou, China.
- The athletes also need a good amount of rest to showcase their maximum sporting potential.
- COVID-appropriate behaviors like sanitizing hands, masking the face, and maintaining the necessary social distance) at the venue may strictly be ensured.

Dear Editor,

The World University Games 2023 (28 July–8 August 2023) under the banner of the International University Sports Federation (FISU) flagged off last Friday in Chengdu, China, is ongoing as the article is drafted. Begun in 1959, christened then as Universiade, this biennial sports carnival is the 31st edition. Scheduled earlier to be held in 2021, it was postponed to this day due to the rampaging COVID-19 (coronavirus disease 2019) then. It attracts thousands of student-athletes, making it larger and more prestigious. Meant for university students from across the globe, this mega event is amongst the largest multi-sport events in the world, both from the number of events and the participants’ numbers points of view. This 12-day-long World University Games incorporate educational and cultural aspects alongside sports events. This sports carnival has grown in dimension decade after decade. For instance, the Daegu Summer World University Games in the Republic of Korea in 2003 registered a gathering from 174 nations, and the Kazan 2013 Summer World University Games in Russia attracted 11 759 participants. This states the likely gathering at the venue this time, too, which has the participation of athletes from more than 110 countries.

The Asian Games 2023 will ensue about a couple of months later, from 23 September to 8 October 2023, at the Zhejiang University of Technology, Hangzhou, China. Originally scheduled between 10 and 25 September, this mega continental event, the 19th Asian Games Hangzhou 2022, was shifted from 2022 to 2023 due to the rapidly spreading COVID-19, similar to the fate of the FISU University Games. Both these sports carnivals are truly global in nature, being held in a country that is high on population and sports enthusiasts, and is still on high alert on health grounds. The still active and evolving SARS-COV-2 (severe acute respiratory syndrome coronavirus 2) variants and subvariants, unless addressed well, are a community health concern. Reports recently opined that infection cycles are very likely in China every sixth month as China has lifted the COVID-19 curbs while the contagious variants are still active^1^. China witnessed a similar infection surge in December 2022 after it relaxed the ‘zero-COVID’ policy^2^, even though one might have acquired immunity from the recent infections. An Indian athlete participating in the FISU had a fever on arrival and was provided with essential medical support with basic quarantine. Fortunately, no SARS-COV-2 virus was detected, and he was declared fit to participate in the FISU events thereafter.

As surveillance efforts across the world are dismantled, genome surveillance is crucial to track novel ‘variants of concern’ (VOCs), if any^3^. Two Omicron subvariants, BA.5.2 and BF.7, were confirmed as prevailing in November–December 2022 through genome analyses of local Beijing samples^2^. The XBB.1.5 and XBB.1.16 are currently being tracked by the WHO as variants of interest (VOIs), and BA.2.75, CH.1.1, XBB, XBB.1.9.1, XBB.1.9.2, and XBB.2.3 are being monitored as variants under monitoring (VUMs). Eighteen thousand new Southeast Asia cases are reported between 5 June and 2 July 2023 recently. Thailand and Bangladesh reported the highest cases, however, with fewer hospital and ICU admissions and, heartening, reduced reported deaths.

The novel subvariants infecting the old, the immunocompromised, or even the vaccinated are concerning. The Chinese strategies to tackle the COVID-19 pandemic seem not to be working on numerous counts, from a possible less-effective vaccination drive to the ‘zero-COVID’ policy (which might not have adequately allowed the attainment of herd immunity). China is aging with a larger population of the elderly, the necessary booster dosage many of whom did not receive. In a never-ending pandemic scenario, mega sports carnivals like the FISU could pose a real healthcare threat due to mass ‘crazy’ and ‘mad’ gatherings that could catalyze the spread of not only COVID-19 but also other such transmissible viruses^4,5^. This calls for meticulous healthcare planning, disease management, and seamless risk monitoring and assessment strategies to avoid untoward healthcare issues, which the Chinese administration has meticulously executed by and large. Random screening, testing, and possibly booster dose for international travelers seem vital. Any adverse occurrence at the venue may have major healthcare pressure with global consequences. Keeping a watch on the urban water supply and sewerage for possible early signs is essential. To nip the healthcare challenge at the bud, the global community could be alerted through electronic and social media by the Chinese authorities attuned to global agencies. Further, COVID-appropriate behavior like sanitizing hands, masking the face, and maintaining the necessary social distance at the venue may strictly be ensured.

Alongside SARS-CoV-2 variants BQ.1 and BA.2.75, the XBB.1.9 with mutated F486P spike protein was identified in China recently during January and June 2023, as per the GISAID database. These could have severe health implications for the elderly, the non-vaccinated, and the immunity-wise fragile individuals^6,7^. Thus, from a health perspective, the events need to be handled with great care and caution. Nevertheless, country-wise healthcare facilities, with extended well-equipped hospital care options in case of an emergency, have been extended at the FISU venue to attend to the health issues. The teenage participants of the FISU University Games are less likely to follow COVID-appropriate behavior unless strictly imposed on them. Thus, the global spreading of the variants and subvariants imported by them could be of concern in their home countries as they disperse from the host country.

Mega sports events play a crucial role in driving the sports economy, tourism, catering, logistics, etc. However, in light of the upcoming Asian Games in the same country in about a couple of months, there could be a few suggestions to revamp the prevailing situation and possibly make it an experience for the stakeholders to cherish. The food, which is not continental in the true sense, seems to be an issue. Although well-hygienic conditions have been maintained, the food preparation and type are wanting (Fig. 1). As a result, players from elsewhere would not relish the food or even might stay hungry. It could be taken care of for the sake of participants and the country’s reputation in the upcoming Asian Games. The other issue is that sports venues in this ongoing FISU are distantly located, many of which are more than 40–50 km away from each other. For this, the athletes are forced to travel for about a couple of hours, which consumes a lot of time to reach the venue, killing their performance spirit. The athletes also need a good amount of rest to showcase their maximum sporting potential. Thus, the issue of the distance from the village to the venue remains critical. The sports facility at KIIT University, Bhubaneswar, is a case example from this perspective. Games need to be planned and arranged in a close satellite point that reduces discomfiture or restlessness among the athletes and officials. The athletes and officials at the ongoing University Games, who have seen and experienced, are mentioning the sports facilities there at KIIT that were available at almost walking distance from the event venue to the place where the players are accommodated. Could it be safely stated that the KIIT University model could be emulated while organizing big international multi-sports carnivals such as that of the FISU.

**Figure 1:**
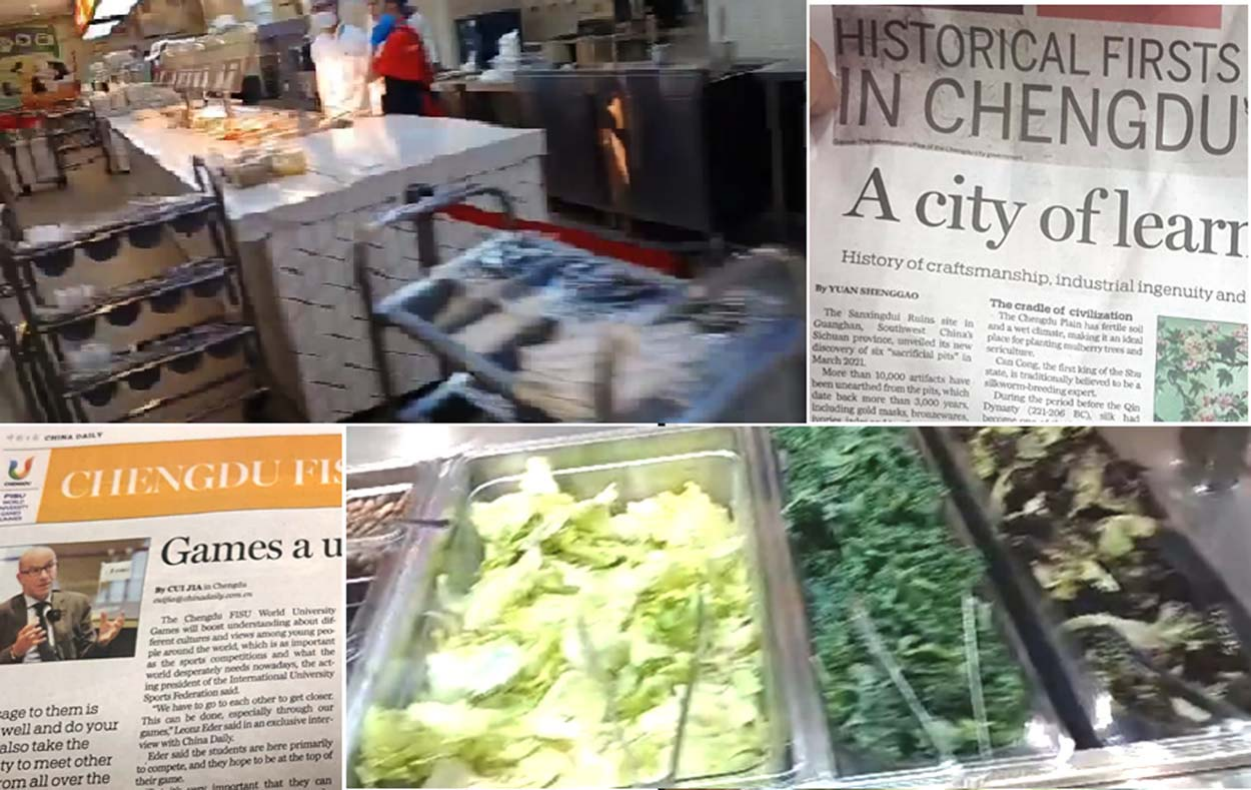
The glimpse of the food, hygiene, and Games village setting of FISU-2023 from ground-zero and the local print media coverage.

## Ethics approval

Ethics approval was not required for this editorial article.

## Consent

Informed consent was not required for this editorial article.

## Sources of funding

No funding was received.

## Author contribution

All authors have contributed significantly.

## Conflicts of interest disclosure

The authors declare no conflict of interest, financial or otherwise.

## Research registration unique identifying number (UIN)

Not applicable.

## Guarantor

Ranjit Sah (corresponding author) is taking full responsibility for the work and/or the conduct of the study, had access to the data, and controlled the decision to publish.

## Data availability statement

All data used to support the findings of this study are included in the article.

## Provenance and peer review

Not commissioned, externally peer-reviewed.
